# Serotype Distribution, Antimicrobial Resistance, and Class 1 Integrons Profiles of *Salmonella* from Animals in Slaughterhouses in Shandong Province, China

**DOI:** 10.3389/fmicb.2017.01049

**Published:** 2017-06-21

**Authors:** Xiaonan Zhao, Chaoqun Ye, Weishan Chang, Shuhong Sun

**Affiliations:** College of Animal Science and Technology, Shandong Agricultural UniversityTai'an, China

**Keywords:** *Salmonella*, animal slaughterhouses, antimicrobial resistance, class 1 integrons, MLST/ST

## Abstract

The current study aimed to analyze the prevalence and characterization of *Salmonella* enterica isolated from animals in slaughterhouses before slaughter. A total of 143 non-duplicate *Salmonella* were recovered from 1,000 fresh fecal swabs collected from four major pig slaughterhouses (49/600, 8.2%) and four major chicken slaughterhouses (94/400, 23.5%) between March and July 2016. Among *Salmonella* isolates from pigs, the predominant serovars were *Salmonella* Rissen (28/49, 57.1%) and Typhimurium (14/49, 28.6%), and high antimicrobial resistance rates were observed for tetracycline (44/49, 89.8%) and ampicillin (16/49, 32.7%). Class 1 integrons were detected in 10.2% (5/49) of these isolates and all contained gene cassettes *aadA2* (0.65 kb). Two β-lactamase genes were detected among these isolates, and most of these isolates carried *bla*_TEM-1_ (46/49), followed by *bla*_OXA-1_(4/49). Seven STs (MLST/ST, multilocus sequence typing) were detected in these isolates, and the predominant type was ST469 (19.6%). Among *Salmonella* isolates from chickens, the predominant serovars were *Salmonella* Indiana (67/94, 71.3%) and Enteritidis (23/94, 24.5%), and high antimicrobial resistance rates were observed for nalidixic acid (89/94, 94.7%), ampicillin (88/94, 93.6%) and tetracycline (81/94, 86.2%). Class 1 integrons were detected in 23 isolates (23/94, 24.5%), which contained empty integrons (0.15 kb, *n* = 6) or gene cassettes *drfA17-aadA5* (1.7 kb, *n* = 6), *aadA2* (1.2 kb, *n* = 5), *drfA16-bla*_PSE-1_*-aadA2-ereA2* (1.6 kb, *n* = 5) or *drfA1-aadA1* (1.4 kb, *n* = 1). Three β-lactamase genes were detected, and all 94 isolates carried *bla*_TEM-1_, followed by *bla*_CTX-*M*-55_ (*n* = 19) and *bla*_SPE−1_ (*n* = 3). Five STs were found in these isolates, and the predominant type was ST17 (71.3%). Our findings indicated that *Salmonella* was widespread in animals at slaughter and may be transmitted from animal to fork.

## Introduction

*Salmonella* enterica is a major global foodborne pathogen (Chiu et al., 2010; Scallan et al., 2011). More than 2,600 different serovars have been identified worldwide (Guibourdenche et al., 2010). In China, *Salmonella* causes an estimated 22.2% of foodborne diseases, and the majority of diseases are associated with the ingestion of contaminated meat products (Wang et al., 2007). Both pigs and chickens have been recognized as an important reservoir for antibiotic resistant *Salmonella*, and the resistance genes can be transferred to other bacteria via mobile genetic elements, such as plasmids and gene cassettes (Vo et al., 2006).

Agents of different antimicrobial classes, such as β-lactams or fluoroquinolones, are frequently used in clinical practice for *Salmonella enterica* infections. Unfortunately, *Salmonella* has gradually developed a high resistance rate to these antimicrobials, leading to the increase of healthcare costs and even clinical treatment failure (Cui et al., 2009; Gonzalez-Sanz et al., 2009). To date, numerous studies have been conducted to monitor antimicrobial resistance and molecular epidemiology of *Salmonella* isolated from pigs and chickens in slaughterhouses (Van et al., 2013; Mohamed et al., 2014).

However, little information concerning prevalence and characterization of *Salmonella* from animals in slaughterhouses in China is available. Shandong province, as a major breeding region, is the main producer of meat products in China. Therefore, major animal slaughterhouses in Shandong province, China were singled out as sampling sites to analyze the prevalence and characterization of *Salmonella* from animals in slaughterhouses.

## Materials and methods

### Description of sampling sites

From March to July 2016, 1,000 fresh fecal swabs were collected from four major pig slaughterhouses with process capacity of 1,500–2,500 pigs per day (150 samples per pig slaughterhouse) and four major chicken slaughterhouses with process capacity of 10,000–40,000 chickens per day (100 samples per chicken slaughterhouse). The animal slaughterhouses are respectively located in Weihai, Ciyao, Zhucheng, and Yantai regions in Shandong province, China. Sampling was carried out before slaughter, and at the time animals from different farms has been mixed.

### Identification and sreotyping of *Salmonella*

From each animal slaughterhouse, fresh fecal swabs were randomly collected from different individual animals, and transported in an ice box to our laboratory within 6 h for further bacteriological analysis. Each swab sample was added into 50 mL buffered peptone water (BPW) and was incubated at 37°C for 16 to 18 h. After that, 0.1 mL of the BPW suspensions was sub-cultured in 10 mL subpackaged Rappaport-Vassiliadis (RV) broth at 42°C for 24 h. One loopful of each RV broth culture was then plated onto xylose lysine tergitol 4 agar plates, and was incubated at 37°C for 24 to 48 h (Yan et al., 2010). Presumptive *Salmonella* colonies were identified using both the VITEK system (BioMerieux, Marcy 1'Etoile, France) and polymerase chain reaction (PCR) amplification of the inherent gene *invA* (Malorny et al., 2003).

All *Salmonella* isolates were serotyped according to the Kauffmann-White scheme by slide agglutination with O and H antigen-specific sera (Tianrun Bio-Pharmaceutical, Ningbo, China) (Grimont and Weill, 2007).

### Antimicrobial susceptibility testing

The Kirby-Bauer disk diffusion method was used in this study to examine resistance of *Salmonella* to 10 commonly used antibiotics, including amoxicillin/clavulanic acid (AMC, 20/10 μg), ampicillin (AMP, 10 μg), cefotaxime (CTX, 30 μg), ciprofloxacin (CIP, 5 μg), florfenicol (FFC, 30 μg), gentamicin (GEN, 10 μg), nalidixic acid (NAL, 10 μg), spectinomycin (SPT, 10 μg), tetracycline (TET, 30 μg), and sulfamethoxazole/trimethoprim (SXT, 1.25/23.75 μg). *Escherichia coli* (ATCC25922) was used as a quality control. The results were interpreted based on the Clinical and Laboratory Standards Institute (CLSI) standards guidelines (CLSI, 2013). *Salmonella* isolates resistant to more than three classes of antimicrobials were defined as multidrug resistance (MDR) isolates.

### Detection of class I integrons and β-lactamase-encoding genes

Bacterial DNA was extracted using a TIANamp bacteria DNA kit (Tiangen, Beijing, China) according to the manufacturer's instructions. The gene cassettes within the variable region of class I integrons were detected via polymerase chain reaction (PCR), using previously described primers and procedures (Kerrn et al., 2002). The PCR products were cloned into the pMD18-T vector using the pMD18-T cloning kit (Takara, Dalian, China) and submitted for sequencing (Invitrogen, Beijing, China).

PCR screening for β-lactamase-encoding genes *bla*_TEM_, *bla*_PSE-1_, *bla*_CMY-2_, *bla*_SHV_, *bla*_DHA-1_, *bla*_OXA_, and *bla*_CTX-*M*_ was performed as previously described (Guerra et al., 2001; Chen et al., 2004; Batchelor et al., 2005; Hasman et al., 2005; Li et al., 2013). The PCR products were purified and subsequently sequenced.

### MLST

The MLST analysis was performed by sequencing the fragments of seven housekeeping genes (*aroC, dnaN, hemD, hisD, purE, sucA*, and *thrA*), and the alleles and STs were assigned according to the MLST scheme at http://mlst.warwick.ac.uk/mlst/dbs/Senterica. A minimum spanning tree was created using Bionumerics software 6.5 (Applied Maths, Kortrijk, Belgium), according to the instructions (the unweighted pair group method of arithmetic averages method).

### Statistical analyses

All statistical analyses were performed using package SPSS 15.0 (SPSS Inc., Chicago, IL, USA). The *chi-square* test was used to compare the prevalence, multidrug resistance rate and carriage of class 1 integron of *Salmonella* isolated from pigs and chickens, and *P* < 0.05 was considered difference significant.

## Results

### Prevalence and serotypes of *Salmonella*

From pig slaughterhouses, 49 *Salmonella* isolates were recovered (49/600, 8.2%), including 13 from Weihai (13/150, 8.7%), 9 from Ciyao (9/150, 6.0%), 11 from Yantai (11/150, 10.7%), and 16 from Zhucheng (16/150, 10.7%) (Table 1). In terms of isolation rate of *Salmonella*, no significant difference was found between the pig slaughterhouses (*P* > 0.05). The 49 *Salmonella* belonged to 6 serovars, including *Salmonella* Rissen (*n* = 28), Typhimurium (*n* = 14), Grampian (*n* = 3), Derby (*n* = 2), Indiana (*n* = 1), and Enteritidis (*n* = 1). The most common serovars were *Salmonella* Rissen (28/49, 57.1%) and Typhimurium (14/49, 28.6%) (Table 2).

**Table 1:** Prevalence of *Salmonella* isolates from pigs and chickens in slaughterhouses.

**Pigs**			**Chickens**	
**Locations**	**No. of samples**	**No. of positive samples**	**No. of samples**	**No. of positive samples**
Weihai	150	13 (8.7%)	100	23 (23.0%)
Ciyao	150	9 (6.0%)	100	33 (33.0%)
Yantai	150	11 (7.3%)	100	17 (17.0%)
Zhucheng	150	16 (10.7%)	100	21 (21.0%)
Total	600	49 (8.2%)	400	94 (23.5%)

**Table 2:** Resistance phenotype, incidence of class 1 integron, and resistance gens in Salmonella isolated from animals in slaughterhouses.

**No**.	**Location**	**Slaughterhouse**	**Serovar**	**Resistance phenotype**	**Integrons/resistance genes**
1	Weihai	Pig	*S*. Typhimurium	AMP, TET	*bla*_TEM-1_
2	Weihai	Pig	*S*. Typhimurium	AMP, TET	*bla*_TEM-1_
3	Weihai	Pig	*S*. Typhimurium	AMP, TET	*bla*_TEM-1_
4	Weihai	Pig	*S*. Typhimurium	AMP, TET	*bla*_TEM-1_
5	Weihai	Pig	*S*. Enteritidis	AMP, GEN, NAL	Class 1 (*aadA2*), *bla*_TEM-1_,
6	Weihai	Pig	*S*. Typhimurium	AMP, TET	*bla*_TEM-1_
7	Weihai	Pig	*S*. Derby	AMP, TET	*bla*_TEM-1_
8	Weihai	Pig	*S*. Rissen	TET	*bla*_TEM-1_
9	Weihai	Pig	*S*. Rissen	TET	*bla*_TEM-1_
10	Weihai	Pig	*S*. Derby	TET	*bla*_TEM-1_
11	Weihai	Pig	*S*. Rissen	TET	*bla*_TEM-1_
12	Weihai	Pig	*S*. Typhimurium	AMP, TET	*bla*_TEM-1_, *bla*_OXA-1_
13	Weihai	Pig	*S*. Typhimurium	TET	*bla*_TEM-1_
14	Ciyao	Pig	*S*. Typhimurium	AMP, TET	*bla*_TEM-1_, *bla*_OXA-1_
15	Ciyao	Pig	*S*. Typhimurium	AMP, TET	*bla*_TEM-1_
16	Ciyao	Pig	*S*. Typhimurium	AMP, TET	*bla*_TEM-1_
17	Ciyao	Pig	*S*. Grampian	TET	*bla*_TEM-1_
18	Ciyao	Pig	*S*. Indiana	CIP, FFC, NAL, SXT, TET	*bla*_TEM-1_, *bla*_OXA-1_
19	Ciyao	Pig	*S*. Grampian	AMP, FFC, SPT, SXT, TET	Class 1 (*aadA2*), *bla*_TEM-1_,
20	Ciyao	Pig	*S*. Rissen	TET	*bla*_TEM-1_
21	Ciyao	Pig	*S*. Grampian	TET	*bla*_TEM-1_
22	Ciyao	Pig	*S*. Typhimurium	AMP, GEN, FFC, NAL, SPT, SXT, TET	Class 1 (*aadA2*), *bla*_TEM-1_
23	Yantai	Pig	*S*. Typhimurium	AMP, GEN, FFC, SPT, SXT, TET	*bla*_TEM-1_, *bla*_OXA-1_
24	Yantai	Pig	*S*. Typhimurium	AMP, GEN, FFC, NAL, SPT, SXT, TET	Class 1 (*aadA2*), *bla*_TEM-1_
25	Yantai	Pig	*S*. Typhimurium	AMP, GEN, FFC, NAL, SPT, SXT, TET	Class 1 (*aadA2*), *bla*_TEM-1_,
26	Yantai	Pig	*S*. Rissen	GEN, TET	*bla*_TEM-1_
27	Yantai	Pig	*S*. Rissen	TET	*bla*_TEM-1_
28	Yantai	Pig	*S*. Rissen		
29	Yantai	Pig	*S*. Rissen	TET	*bla*_TEM-1_
30	Yantai	Pig	*S*. Rissen	TET	*bla*_TEM-1_
31	Yantai	Pig	*S*. Rissen		
32	Yantai	Pig	*S*. Rissen	TET	*bla*_TEM-1_
33	Yantai	Pig	*S*. Rissen	GEN, TET	*bla*_TEM-1_
34	Zhucheng	Pig	*S*. Rissen	TET	*bla*_TEM-1_
35	Zhucheng	Pig	*S*. Rissen	TET	*bla*_TEM-1_
36	Zhucheng	Pig	*S*. Rissen	TET	*bla*_TEM-1_
37	Zhucheng	Pig	*S*. Rissen	TET	*bla*_TEM-1_
38	Zhucheng	Pig	*S*. Rissen	TET	*bla*_TEM-1_
39	Zhucheng	Pig	*S*. Rissen	TET	*bla*_TEM-1_
40	Zhucheng	Pig	*S*. Rissen		
41	Zhucheng	Pig	*S*. Rissen	TET	*bla*_TEM-1_
42	Zhucheng	Pig	*S*. Rissen	TET	*bla*_TEM-1_
43	Zhucheng	Pig	*S*. Rissen	TET	*bla*_TEM-1_
44	Zhucheng	Pig	*S*. Rissen	TET	*bla*_TEM-1_
45	Zhucheng	Pig	*S*. Rissen	TET	*bla*_TEM-1_
46	Zhucheng	Pig	*S*. Rissen		*bla*_TEM-1_
47	Zhucheng	Pig	*S*. Rissen	TET	*bla*_TEM-1_
48	Zhucheng	Pig	*S*. Rissen	TET	*bla*_TEM-1_
49	Zhucheng	Pig	*S*. Rissen	TET	*bla*_TEM-1_
50	Weihai	Chicken	*S*. Enteritidis	AMP, CTX, NAL	*bla*_TEM-1_
51	Weihai	Chicken	*S*. Indiana	AMP, CIP, CTX, NAL, TET	*bla*_TEM-1_
52	Weihai	Chicken	*S*. Enteritidis	AMP, GEN, CTX, FFC, NAL, TET	Class 1 (*aadA2*), *bla*_TEM-1_
53	Weihai	Chicken	*S*. Enteritidis	AMP, GEN, CTX, FFC, NAL, TET	*bla*_TEM-1_, *bla*_CTX-M-55_
54	Weihai	Chicken	*S*. Enteritidis	AMP, GEN, CTX, FFC, NAL, TET	*bla*_TEM-1_, *bla*_CTX-*M*-55_
55	Weihai	Chicken	*S*. Enteritidis	AMP, GEN, CTX, FFC, NAL, TET	*bla*_TEM-1_, *bla*_CTX-*M*-55_
56	Weihai	Chicken	*S*. Indiana	AMP, CTX, NAL	Class 1 (*drfA1*-*aadA1*), *bla*_TEM-1_
57	Weihai	Chicken	*S*. Enteritidis	AMP, GEN, CTX, FFC, NAL, TET	*bla*_TEM-1_, *bla*_CTX-*M*-55_
58	Weihai	Chicken	*S*. Typhimurium	AMP, GEN, SPT	Class 1 (*aadA2*), *bla*_TEM-1_
59	Weihai	Chicken	*S*. Typhimurium	AMP, SPT	*bla*_TEM-1_
60	Weihai	Chicken	*S*. Enteritidis	AMP, GEN, CTX, FFC, NAL, TET	*bla*_*TEM*−1_, *bla*_CTX-*M*-55_
61	Weihai	Chicken	*S*. Enteritidis	AMP, CTX, NAL	*bla*_TEM-1_
62	Weihai	Chicken	*S*. Enteritidis	AMP, GEN, CTX, FFC, NAL, TET	*bla*_TEM-1_
63	Weihai	Chicken	*S*. Enteritidis	AMP, CTX, FFC, NAL, TET	*bla*_TEM-1_, *bla*_CTX-*M*-55_
64	Weihai	Chicken	*S*. Enteritidis	AMP, GEN, CTX, FFC, NAL, TET	Class 1 (*drfA17-aadA5*), *bla*_TEM-1_, *bla*_CTX-*M*-55_
65	Weihai	Chicken	*S*. Enteritidis	AMP, GEN, CTX, FFC, NAL, TET	*bla*_TEM-1_
66	Weihai	Chicken	*S*. Enteritidis	AMP, GEN, CTX, FFC, NAL, TET	Class 1 (*drfA17-aadA5*), *bla*_TEM-1_
67	Weihai	Chicken	*S*. Enteritidis	AMP, GEN, CTX, FFC, NAL, TET	*bla*_TEM-1_, *bla*_CTX-*M*-55_
68	Weihai	Chicken	*S*. Typhimurium	AMP, SPT	Class 1 (*aadA2*), *bla*_TEM-1_
69	Weihai	Chicken	*S*. Enteritidis	NAL	*bla*_TEM-1_
70	Weihai	Chicken	*S*. Enteritidis	AMP, GEN, CTX, FFC, NAL, TET	*bla*_TEM-1_, *bla*_CTX-*M*-55_
71	Weihai	Chicken	*S*. Enteritidis	AMP, CIP, CTX, NAL, TET	*bla*_TEM-1_
72	Weihai	Chicken	*S*. Enteritidis	AMP, CTX, NAL	empty integron, *bla*_TEM-1_
73	Ciyao	Chicken	*S*. Indiana	AMP, CTX, NAL	*bla*_TEM-1_
74	Ciyao	Chicken	*S*. Enteritidis	NAL	*bla*_TEM-1_
75	Ciyao	Chicken	*S*. Indiana	AMP, NAL, TET	*bla*_TEM-1_
76	Ciyao	Chicken	*S*. Indiana	AMP, CIP, NAL, TET	*bla*_TEM-1_, *bla*_CTX-*M*-55_
77	Ciyao	Chicken	*S*. Enteritidis		*bla*_TEM-1_
78	Ciyao	Chicken	*S*. Indiana	AMP, CIP, NAL, TET	*bla*_TEM-1_
79	Ciyao	Chicken	*S*. Indiana	AMP, NAL, TET	*bla*_TEM-1_
80	Ciyao	Chicken	*S*. Indiana	AMP, NAL, TET	*bla*_TEM-1_
81	Ciyao	Chicken	*S*. Indiana	AMP, CIP, NAL, TET	*bla*_TEM-1_
82	Ciyao	Chicken	*S*. Enteritidis	AMP, CIP, NAL, TET	Class 1 (*drfA17-aadA5*), *bla*_TEM-1_
83	Ciyao	Chicken	*S*. Indiana	AMP, CIP, NAL, TET	*bla*_TEM-1_
84	Ciyao	Chicken	*S*. Indiana	AMP, CIP, NAL, TET	*bla*_TEM-1_
85	Ciyao	Chicken	*S*. Indiana	AMP, NAL, TET	*bla*_TEM-1_
86	Ciyao	Chicken	*S*. Indiana	AMP, CIP, NAL, TET	*bla*_TEM-1_, *bla*_CTX-*M*-55_
87	Ciyao	Chicken	*S*. Indiana	AMP, CIP, NAL, TET	*bla*_TEM-1_
88	Ciyao	Chicken	*S*. Indiana	AMP, NAL, TET	*bla*_TEM-1_
89	Ciyao	Chicken	*S*. Indiana	AMP, CIP, NAL, TET	*bla*_TEM-1_
90	Ciyao	Chicken	*S*. Indiana	AMP, CIP, GEN, NAL, TET	Class 1 (*drfA16*-*bla*_*PSE-1*_-*aadA2*-*ereA2), bla*_TEM-1_, *bla*_SPE-1_
91	Ciyao	Chicken	*S*. Indiana	AMP, CIP, NAL, TET	*bla*_TEM-1_
92	Ciyao	Chicken	*S*. Indiana	AMP, CIP, NAL, TET	*bla*_TEM-1_
93	Ciyao	Chicken	*S*. Indiana	AMP, CIP, NAL, TET	*bla*_TEM-1_
94	Ciyao	Chicken	*S*. Indiana	AMP, CIP, NAL, TET	*bla*_TEM-1_
95	Ciyao	Chicken	*S*. Indiana	AMP, CIP, NAL, TET	*bla*_TEM-1_
96	Ciyao	Chicken	*S*. Indiana	AMP, CIP, NAL, TET	*bla*_TEM-1_
97	Ciyao	Chicken	*S*. Indiana	AMP, NAL, TET	*bla*_TEM-1_
98	Ciyao	Chicken	*S*. Indiana	AMP, CIP, CTX, NAL, TET	Class 1 (*aadA2*), *bla*_TEM-1_, *bla*_CTX-*M*-55_
99	Ciyao	Chicken	*S*. Indiana	AMP, CIP, NAL, TET	*bla*_TEM-1_
100	Ciyao	Chicken	*S*. Indiana	AMP, CIP, NAL, TET	empty integron, *bla*_TEM-1_
101	Ciyao	Chicken	*S*. Indiana	AMP, CIP, NAL, TET	*bla*_TEM-1_
102	Ciyao	Chicken	*S*. Indiana	AMP, CIP, NAL, TET	*bla*_TEM-1_
103	Ciyao	Chicken	*S*. Indiana	AMP, CIP, CTX, NAL, TET	Class 1 (*drfA16*-*bla*_*PSE-1*_-*aadA2*-*ereA2*), *bla*_TEM-1_
104	Ciyao	Chicken	*S*. Indiana	AMP, CIP, CTX, NAL, TET	*bla*_TEM-1_, *bla*_CTX-*M*-55_
105	Ciyao	Chicken	*S*. Indiana	AMP, CIP, CTX, NAL, TET	*bla*_TEM-1_, *bla*_CTX-*M*-55_
106	Yantai	Chicken	*S*. Indiana	AMP, CIP, CTX, NAL, TET	empty integron, *bla*_TEM-1_
107	Yantai	Chicken	*S*. Hadar	NAL, TET	*bla*_TEM-1_
108	Yantai	Chicken	*S*. Indiana	AMP, CIP, CTX, NAL, TET	*bla*_TEM-1_, *bla*_CTX-*M*-55_
109	Yantai	Chicken	*S*. Indiana	AMP, CIP, CTX, NAL, TET	Class 1 (*drfA17-aadA5*), *bla*_TEM-1_
110	Yantai	Chicken	*S*. Indiana	AMP, CIP, CTX, NAL, TET	*bla*_TEM-1_, *bla*_CTX-*M*-55_
111	Yantai	Chicken	*S*. Indiana	AMP, CIP, CTX, NAL, TET	*bla*_TEM-1_, *bla*_CTX-*M*-55_
112	Yantai	Chicken	*S*. Indiana	AMP, CIP, NAL, TET	*bla*_TEM-1_
113	Yantai	Chicken	*S*. Indiana	AMP, CIP, NAL, TET	*bla*_TEM-1_
114	Yantai	Chicken	*S*. Indiana	AMP, CIP, CTX, NAL, TET	*bla*_TEM-1_, *bla*_CTX-*M*-55_
115	Yantai	Chicken	*S*. Indiana	AMP, CIP, NAL, TET	*bla*_TEM-1_
116	Yantai	Chicken	*S*. Indiana	AMP, CIP, GEN, CTX, NAL, TET	Class 1 (*drfA17-aadA5*), *bla*_TEM-1_
117	Yantai	Chicken	*S*. Indiana	NAL	*bla*_TEM-1_
118	Yantai	Chicken	*S*. Indiana	AMP, CIP, CTX, NAL, TET	Class 1 (*drfA16*-*bla*_*PSE-1*_-*aadA2*-*ereA2*), *bla*_TEM-1_, *bla*_CTX-*M*-55_
119	Yantai	Chicken	*S*. Indiana	AMP, CIP, NAL, TET	*bla*_TEM-1_
120	Yantai	Chicken	*S*. Indiana	AMP, CIP, NAL, TET	*bla*_TEM-1_
121	Yantai	Chicken	*S*. Indiana	AMP, CIP, NAL, TET	empty integron, *bla*_TEM-1_
122	Yantai	Chicken	*S*. Indiana	AMP, CIP, NAL, TET	*bla*_TEM-1_
123	Zhucheng	Chicken	*S*. Indiana	AMP, CIP, NAL, TET	Class 1 (*drfA16*-*bla*_*PSE-1*_-*aadA2*-*ereA2*), *bla*_TEM-1_, *bla*_SPE-1_
124	Zhucheng	Chicken	*S*. Indiana	AMP, CIP, NAL, TET	*bla*_TEM-1_
125	Zhucheng	Chicken	*S*. Enteritidis		*bla*_TEM-1_
126	Zhucheng	Chicken	*S*. Indiana	AMP, CIP, NAL, TET	*bla*_TEM-1_
127	Zhucheng	Chicken	*S*. Indiana	AMP, CIP, NAL, TET	*bla*_TEM-1_
128	Zhucheng	Chicken	*S*. Indiana	AMP, CIP, NAL, TET	*bla*_TEM-1_
129	Zhucheng	Chicken	*S*. Enteritidis	AMP, CIP, NAL, TET	Class 1 (*aadA2*), *bla*_TEM-1_
130	Zhucheng	Chicken	*S*. Indiana	AMP, CIP, NAL, TET	*bla*_TEM-1_
131	Zhucheng	Chicken	*S*. Indiana	AMP, CIP, NAL, TET	*bla*_TEM-1_
132	Zhucheng	Chicken	*S*. Indiana	AMP, CIP, NAL, TET	*bla*_TEM-1_
133	Zhucheng	Chicken	*S*. Indiana	AMP, CIP, NAL, TET	*bla*_TEM-1_
134	Zhucheng	Chicken	*S*. Indiana	AMP, CIP, NAL, TET	*bla*_TEM-1_
135	Zhucheng	Chicken	*S*. Indiana	AMP, CIP, CTX, NAL, TET	Class 1 (*drfA16*-*bla*_*PSE-1*_-*aadA2*-*ereA2*), *bla*_TEM-1_, *bla*_SPE-1_
136	Zhucheng	Chicken	*S*. Indiana	AMP, CIP, NAL, TET	*bla*_TEM-1_
137	Zhucheng	Chicken	*S*. Indiana	AMP, CIP, NAL, TET	empty integron, *bla*_TEM-1_
138	Zhucheng	Chicken	*S*. Indiana	AMP, CIP, NAL, TET	empty integrons, *bla*_TEM-1_
139	Zhucheng	Chicken	*S*. Indiana	AMP, CIP, NAL, TET	*bla*_TEM-1_
140	Zhucheng	Chicken	*S*. Indiana	AMP, CIP, GEN, NAL, TET	Class 1 (*drfA17-aadA5*), *bla*_TEM-1_
141	Zhucheng	Chicken	*S*. Indiana	AMP, CIP, NAL, TET	*bla*_TEM-1_
142	Zhucheng	Chicken	*S*. Indiana	AMP, CIP, GEN, NAL, TET	*bla*_TEM-1_
143	Zhucheng	Chicken	*S*. Indiana	AMP, CIP, GEN, NAL, TET	*bla*_TEM-1_

*amoxicillin/clavulanic acid (AMC), ampicillin (AMP), cefotaxime (CTX), ciprofloxacin (CIP), florfenicol (FFC), gentamicin (GEN), nalidixic acid (NAL), spectinomycin (SPT), tetracycline (TET,) and sulfamethoxazole/trimethoprim (SXT)*.

From chicken slaughterhouses, 94 *Salmonella* isolates were recovered (94/400, 23.5%), including 23 from Weihai (23/100, 23.0%), 33 from Ciyao (33/100, 33.0%), 17 from Yantai (17/100, 17.0%), and 21 from Zhucheng (21/150, 21.0%) (Table 1). In terms of isolation rate of *Salmonella*, no significant difference was found between the chicken slaughterhouses (*P* > 0.05). These 94 *Salmonella* isolates belonged to 4 serovars, including *Salmonella* Indiana (*n* = 67), Enteritidis (*n* = 23), Typhimurium (*n* = 3), and Hadar (*n* = 1). The dominant serovars were *Salmonella* Indiana (67/94, 71.3%) and Enteritidis (23/94, 24.5%) (Table 2).

### Antimicrobial susceptibility testing

All 49 isolates from pig slaughterhouses were susceptible to amoxicillin/clavulanic acid and cefotaxime. But most isolates were resistance to tetracycline (44/49, 89.8%) and ampicillin (16/49, 32.7%). In addition, 7 isolates (7/49, 14.3%) exhibited MDR (Table 2). In addition, 4 isolates were susceptible to all antibiotics used in this study.

All 94 isolates from chicken slaughterhouses were susceptible to amoxicillin/clavulanic acid and sulfamethoxazole/trimethoprim. But most isolates were resistant to nalidixic acid (89/94, 94.7%), ampicillin (87/94, 92.6%), and tetracycline (81/94, 86.2%). Eighty-six isolates (86/94, 91.5%) exhibited MDR (Table 2). Of note, MDR rate of *Salmonella* from chickens was higher than that from pigs (*P* < 0.05). In addition, 2 isolates were susceptible to all antibiotics used in this study.

### Characteristics of class 1 integrons and β-lactamase-encoding genes

Among the 49 isolates recovered from pigs, class 1 integrons were found in 5 isolates (5/49, 10.2%), including 4 *Salmonella* Typhimurium and 1 Enteritidis. The 5 isolates only contained the single resistance gene cassette *aadA2* (0.65 kb). Two β-lactamase genes were detected among the isolates, most of the isolates carried *bla*_SPE-1_ (*n* = 46) and *bla*_OXA-1_(*n* = 4) (Table 2).

Among the 94 isolates recovered from chicken, class 1 integrons were found in 23 isolates (23/94, 24.5%), including 16 *Salmonella* Indiana, 5 Enteritidis and 2 Typhimurium. Of these isolates, 5 groups of resistance gene cassettes were detected: empty integrons (0.15 kb, *n* = 6), *drfA17-aadA5* (1.6 kb, *n* = 6), *aadA2* (1.2 kb, *n* = 5), *drfA16*-*bla*_PSE_-_1_-*aadA2*-*ereA2* (1.7 kb, *n* = 5), and *drfA1*-*aadA1* (1.4 kb, *n* = 1). Three β-lactamase genes were detected among these isolates. Most of the isolates carried *bla*_TEM-1_ (*n* = 94), followed by *bla*_CTX-M-55_ (*n* = 19) and *bla*_SPE-1_(*n* = 3) (Table 2).

### MLST

One hundred and forty-three *Salmonella* isolates were divided into 9 STs, including 7 STs from pigs (ST11, ST17, ST19, ST34, ST40, ST358, and ST469), and 5 STs from chickens (ST11, ST17, ST19, ST33, and ST3172). The STs identified in the present study showed the following correlations with *Salmonella* serovars: ST11 with *Salmonella* Enteritidis, ST17 with Indiana, and ST469 with Rissen.

**Figure 1:**
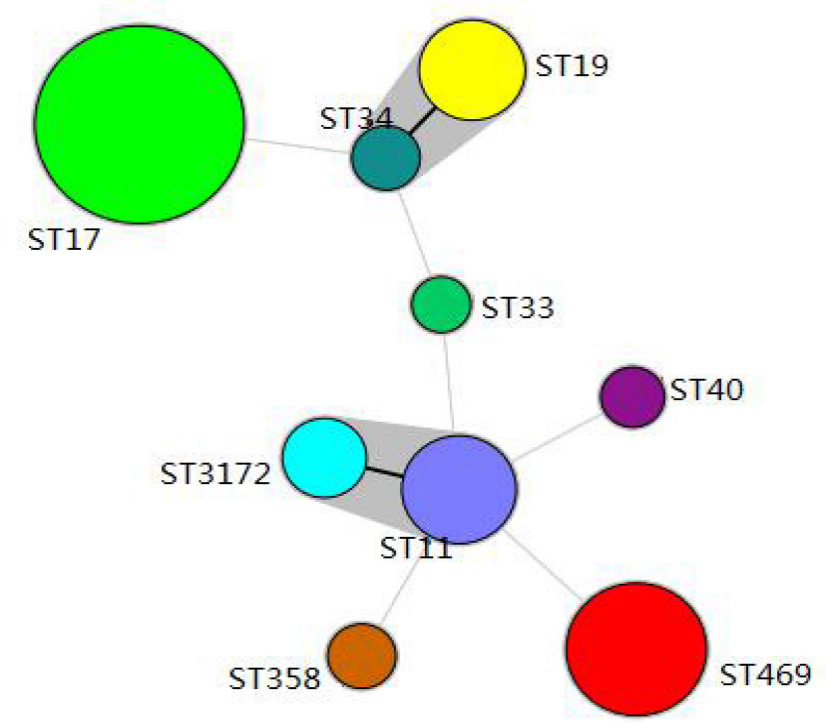
Minimum-spanning tree analysis of the *Salmonella* isolated from pigs and chickens in slaughterhouses. Each circle represents one ST, and the area of the circle corresponds to the number of isolates, the gray region indicates that ST19 and ST34 belong to a clonal complex, ST11, and ST3172 belong to a clonal complex.

BioNumerics software version 6.5 was used to generate a minimum-spanning tree based on all the sources of STs (Figure 1). The dominant ST was ST17 (68/143, 47.6%), with most of isolates from chickens and only one from pigs, followed by ST469 (28/143, 19.6%), with all isolates from pigs. ST34 and ST19 belonged to one clone complex and had the same serovars of *Salmonella* Typhimurium. ST11 and ST3172 belonged to one clone complex, and had the same serovars of *Salmonella* Enteritidis.

## Discussion

In this study, *Salmonella* isolation rate from pigs (8.2%) was much lower than that (71.8%) in Jiangsu province, China (Cai et al., 2016), and the most common serotype in pigs was *Salmonella* Rissen, which in consistent with the result from the retail pork products in Thailand (Prapas et al., 2016). However, this finding was different from that reported in EU in which *Salmonella* Typhimurium was the most common serotype (European Food Safety Authority, 2014). Of note, *Salmonella* Rissen isolates from pigs only showed resistant to tetracycline (85.7%), which may be associated with the fact that the antimicrobial is frequently used in pig farms in China (Bai et al., 2015).

The *Salmonella* isolation rate from chickens (23.5%) was similar to the result reported for frozen chicken meat in Shandong province (26.3%), China (Cui et al., 2016). However, the result in this study was much lower than that (45.2%) from chickens in Henan province, China (Bai et al., 2015) and was higher than that (4.5%) from large-scale chicken farms in Shanghai, China (Liu et al., 2010). The difference of these isolation rates may be related with collection seasons, culture methods, and local environments. In the present study, the most common serotypes identified in chickens were *Salmonella* Indiana and Enteritidis, consistent with findings reported in Henan, China (Bai et al., 2015). However, this finding differed from the result reported in Sichuan province, China, in which *Salmonella* Derby and Typhimurium were the most common serotypes (Li et al., 2013). Additionally, *Salmonella* Kentucky and Enteritidis were the most common serotypes in the USA (National Antimicrobial Resistance Monitoring System, 2011), and *Salmonella* Typhimurium in the EU (European Food Safety Authority, 2014). This difference may be associated with geographical regions. In the present study, *Salmonella* Indiana showed a high MDR rate (61/68, 89.7%), similar with the result conducted in China (Lu et al., 2011), which demonstrated that most of *Salmonella* Indiana showed MDR, and these bacteria were not only resistant to streptomycin and tetracycline but also were resistant to chloramphenicol, fluoroquinolones and cephalosporin antibiotics.

In the current study, most *Salmonella* isolates showed high resistance to tetracycline, ampicillin, and nalidixic acid, similar to the report on slaughterhouses in Italy (Piras et al., 2011), suggesting that these drugs may have been widely used on animals during disease control and prevention. A high resistance rate (63.4%) of nalidixic acid was observed in *Salmonella* isolates, consistent with other reports (Piras et al., 2011; Siriken et al., 2015). The resistance rate to ciprofloxacin was up to 42.7%. The results may be related with the fact that fluoroquinolone antibiotics are the most common treatment for *Salmonella* infections. A relatively high resistance rate to cefotaxime (29.1%) was observed in this study, which may be associated with the fact that third-generation cephalosporins have become the primary drugs for the treatment of salmonellosis because of the increase in fluoroquinolone resistance. In addition, the results of the present study showed the high prevalence of multidrug resistant *Salmonella* isolates in chickens (91.5%), much higher than those reported in Henan province (46.0%), China (Bai et al., 2015) and in central China (34.7%) (Kuang et al., 2015). In this study, MDR isolate rate of *Salmonella* (91.5%) from chickens were higher than that (14.3%) from pigs, and the higher occurrence of MDR *Salmonella* isolates from chickens likely reflects the extensive use of antibiotics during intensive rearing. In addition, MDR *Salmonella* is serotype-dependent (Clemente et al., 2014): the data provided evidence that *Salmonella* Indiana, Typhimurium and Enteritidis were strongly associated with MDR phenotypes. However, these findings were different from a previous study showing that *Salmonella* Derby is commonly associated with MDR (Newell et al., 2010).

In the present study, PCR identified class 1 integrons in 19.6% of *Salmonella* isolates, which was similar to the 15.0% reported from retail meat products in the USA (Zhao et al., 2009) but higher than that of (2.8%) reported from milk products (Van et al., 2013). In the present study, the incidence of class 1 integrons was higher in *Salmonella* from chickens (24.5%) than *Salmonella* from pigs (10.2%) (*P* < 0.05). Class 1 integrons are often associated with MDR *Salmonella* isolates, consistent with the result of the present study. In addition, the *Salmonella* isolates carrying class 1 integrons included *Salmonella* Typhimurium, Enteritidis, and Indiana.

Four β-lactamase genes were detected among *Salmonella* isolates recovered from pigs and chickens: *bla*_TEM-1_, *bla*_PSE-1_, *bla*_OXA-1_, and *bla*_CTX-*M*-55_. Most isolates carried *bl*a_TEM-1_, consistent with the report from meat and milk products in Egypt (Ashraf et al., 2014), but different from the report from animal slaughterhouses and retail meat products in Sichuan, China, which showed the dominant β-lactamase gene was *bla*_OXA-1_, followed by *bla*_TEM-1_, *bla*_PSE-1_, and *bla*_CMY-2_ (Li et al., 2013). The fact that 46 *Salmonella* from pigs carried *bla*_TEM-1_ whereas only 16 were resistant to ampicillin, and only 88 out of 94 *Salmonella* carrying *bla*_TEM-1_ from chickens showed resistant to ampicillin may be associated with the expression status of *bla*_TEM-1_ genes and is needed to be further studied.

In addition, *bla*_CMY-2_ encodes resistance to third-generation cephalosporins, an important class of antibiotics used to treat complicated cases of salmonellosis (Gonzalez-Sanz et al., 2009). The incidence of *bla*_CMY-2_-positive *Salmonella* in China was low and was only reported in Shanxi and Sichuan (Yang et al., 2010; Li et al., 2013).

The MLST results revealed 9 STs identified in *Salmonella* from pigs and chickens. ST19 and ST34 have continually been reported to cause human salmonellosis in recent years, and these bacteria belong to the same serotype, *Salmonella* Typhimurium (Cai et al., 2016), and this circumstance was also true for *Salmonella* Enteritidis, represented by ST11 and ST3172. These findings suggested that serovars and STs were tightly coupled (Sukhnanand et al., 2005). ST358 is rare in China and corresponds to *Salmonella* Grampian, which causes an unusual increase in human cases of *Salmonella* Grampian infections (Horvath et al., 2013). This observation indicates that *Salmonella* could spread from animals to humans via pork and chicken products (Osman et al., 2014).

## Conclusions

Collectively, our findings exhibit the prevalence and characteristics of *Salmonella* isolated from animals in slaughterhouses in Shandong province, China. In addition, this study highlights the necessity to carry out the long-term surveillance for *Salmonella* recovered from food-producing animals.

## Author contributions

WC and SS: conceived and designed the study. XZ and CY: performed the experiments and analyzed the data. XZ, WC, and SS: wrote and revised the manuscript.

### Conflict of interest statement

The authors declare that the research was conducted in the absence of any commercial or financial relationships that could be construed as a potential conflict of interest.
